# Available phosphorus levels modulate gene expression related to intestinal calcium and phosphorus absorption and bone parameters differently in gilts and barrows

**DOI:** 10.5713/ab.22.0251

**Published:** 2022-11-14

**Authors:** Julia Christiane Vötterl, Jutamat Klinsoda, Simone Koger, Isabel Hennig-Pauka, Doris Verhovsek, Barbara U. Metzler-Zebeli

**Affiliations:** 1 Nutritional Physiology, Institute of Physiology, Pathophysiology and Biophysics, Department of Biomedical Sciences, University of Veterinary Medicine Vienna, Vienna 1210, Austria; 2 Institute of Food Research and Product Development, University of Kasetsart, Bangkok 10900, Thailand; 3 Institute of Animal Nutrition and Functional Plant Compounds, Department for Farm Animals and Veterinary Public Health, University of Veterinary Medicine Vienna, Vienna 1210, Austria; 4 Field Station for Epidemiology, University of Veterinary Medicine Hannover, Foundation, Bakum 49456, Germany; 5 University Clinic of Swine, Department for Farm Animals and Veterinary Public Health, University of Veterinary Medicine Vienna, Vienna 1210, Austria

**Keywords:** Bones, Intestines, Kidneys, Phytases, Phosphorus, Serum

## Abstract

**Objective:**

Dietary phytase increases bioavailability of phytate-bound phosphorus (P) in pig nutrition affecting dietary calcium (Ca) to P ratio, intestinal uptake, and systemic utilization of both minerals, which may contribute to improper bone mineralization. We used phytase to assess long-term effects of two dietary available P (aP) levels using a one-phase feeding system on gene expression related to Ca and P homeostasis along the intestinal tract and in the kidney, short-chain fatty acids in stomach, cecum, and colon, serum, and bone parameters in growing gilts and barrows.

**Methods:**

Growing pigs (37.9±6.2 kg) had either free access to a diet without (Con; 75 gilts and 69 barrows) or with phytase (650 phytase units; n = 72/diet) for 56 days. Samples of blood, duodenal, jejunal, ileal, cecal, and colonic mucosa and digesta, kidney, and metacarpal bones were collected from 24 pigs (6 gilts and 6 barrows per diet).

**Results:**

Phytase decreased daily feed intake and average daily gain, whereas aP intake increased with phytase versus Con diet (p<0.05). Gilts had higher colonic expression of *TRPV5*, *CDH1*, *CLDN4*, *ZO1*, and *OCLN* and renal expression of *TRPV5* and *SLC34A3* compared to barrows (p<0.05). Phytase increased duodenal expression of *TRPV5*, *TRPV6*, *CALB1*, *PMCA1b*, *CDH1*, *CLDN4*, *ZO1*, and *OCLN* compared to Con diet (p<0.05). Furthermore, phytase increased expression of *SCL34A2* in cecum and of *FGF23* and *CLDN4* in colon compared to Con diet (p<0.05). Alongside, phytase decreased gastric propionate, cecal valerate, and colonic caproate versus Con diet (p<0.05). Phytase reduced cortical wall thickness and index of metacarpal bones (p<0.05).

**Conclusion:**

Gene expression results suggested an intestinal adaptation to increased dietary aP amount by increasing duodenal trans- and paracellular Ca absorption to balance the systemically available Ca and P levels, whereas no adaption of relevant gene expression in kidney occurred. Greater average daily gain in barrows related to higher feed intake.

## INTRODUCTION

Albeit causes for improper bone mineralization in pigs are multifactorial in nature, inadequate nutrition, like an imbalanced dietary ratio of calcium (Ca) and phosphorus (P), is a major factor [1]. Due to environmental concerns, supplementation of diets with inorganic P decreased over the past decades, greatly increasing the reliance on microbial phytase addition. Phytase improves the bioavailability of P out of plant storage phytate-P, replacing inorganic P in diets. This practice can lower the dietary Ca/available P (aP) ratio affecting the intestinal solubility and mucosal uptake of Ca and P. The intestinal absorption of Ca declines with increased intestinal P availability. However, the long-term effects of modified Ca/aP levels on the systemic availability and utilization of Ca and P for bone development alongside its influence on intestinal and renal gene expression have not been sufficiently studied in fattening pigs. The efficiency of dietary phytase to release P depends on several factors like acidic stomach conditions which are determined by stomach filling based on individual feed intake [2–4]. Attempts have been made to link dietary Ca and P intake with serum values for Ca, P, and regulatory hormones, being influenced by the age and sex of the pig. Overall, at 12 weeks of age, serum P and serum Ca/P ratio, and at 20 weeks of age serum 25-hydroxyvitamin D3 and D2 (VitD) were the most reliable parameters to predict aP intake. Active VitD, parathormone, and bone-synthesized fibroblast growth factor 23 (FGF23) regulate the Ca and P homeostasis via actions in the intestine, kidney, and bone [5,6]. The intestinal absorption of Ca and P comprises two absorption routes. First, active transcellular carrier-mediated absorption takes primarily place in the small intestine. Second, passive paracellular route relies on electrochemical gradients and pores formed through junctional complexes especially tight- and adherens-junction proteins. Active P absorption is enhanced at low dietary P levels and further regulated by VitD and supposedly FGF23. The kidney adjusts circulating Ca and P levels through excretion or reabsorption of Ca and P orchestrated by VitD and FGF23 [7–9]. Differences in dietary aP levels also induce shifts in the microbial communities and their metabolic activity (e.g. short-chain fatty acids [SCFA] production) as P is an essential component for bacterial metabolism. Rising the luminal SCFA increases the mineral solubility and cation absorption by decreasing intestinal pH [10,11]. We hypothesized that long-term feeding of an increased dietary aP amount to growing pigs for 8 weeks would increase P and Ca absorption based on the increased mucosal expression of transport proteins. If Ca and P are absorbed in a favorable ratio, improved bone characteristics may be found with the increased dietary aP. Simultaneously, we hypothesized that systemic regulation either apparent in regulatory parameters in serum or adapted gene expression in the kidney will not be affected by dietary P levels. Furthermore, we assumed increased microbial activity and luminal SCFA due to the enhanced P availability as previously observed [10], which can promote intestinal Ca and P absorption. We used phytase to assess the long-term effects of two different dietary aP levels on gene expression related to Ca and P homeostasis along the intestinal tract and in the kidney as well as regulatory serum parameters and bone metrics in growing gilts and barrows using an one-phase feeding system. For further understanding of the interplay with systemic utilization and microbial activity, we analyzed serum parameters and SCFAs in stomach, cecum and colon.

## MATERIALS AND METHODS

The data presented here were derived from a subset of 24 pigs from the first and two replicate batches of a published study evaluating serum parameters to predict dietary intake of Ca and aP presented in Vötterl et al [5]. The institutional ethics committee of the University of Veterinary Medicine Vienna and the National authority approved all procedures involving animal handling and treatment according to the Law for Animal Experiments, Tierversuchsgesetz (GZ 68.205/ 0227/3b/2018).

### Animals, housing, and experimental design

In the first and second replicate batch of the previously published study Vötterl et al [5], 144 (75 gilts and 69 barrows; 11 weeks of age; 37.9±6.2 kg) fattening pigs (Swiss Large White× Piétrain) from 13 litters were used. Data from this feeding trial were already presented for the entire group of pigs in Vötterl et al [5]. The outdoor climate-fattening unit consisted of six pens for 12 pigs each (3.50 m×5.48 m/pen) with slatted flooring, two nipple drinkers, and one automatic feeding station per pen. Littermates (n = 12/pen) were housed together, if possible six barrows and six gilts. Pigs were allocated to one out of two diets: a control diet without (Con diet; 37 gilts and 35 barrows) and a diet supplemented with phytase (Phy diet; 38 gilts and 34 barrows). Each replicate batch lasted 56 days (eight weeks) with one additional week before the start of the experiment for acclimatization to the pens and feeding system already receiving experimental diets. The body weight (BW) was measured on experimental days 1 and 49. From the 144 pigs, a subsample of 24 pigs, precisely one gilt and one barrow per pen and replicate, were selected for intestinal sampling based on their continuous average daily gain (ADG) as an indicator of good health and their BW on day 49 being close to the mean BW per pen and dietary group.

### Diets, feeding, and growth performance

The two diets (Table 1) were formulated to meet the current recommendations for nutrient requirements except for total P (tP), which was marginally lower than recommended for pigs’ BW at the beginning of the experiment. The Phy diet comprised 650 phytase units (FTU) per kilogram complete feed of a 6-phytase derived from Escherichia coli (VM Phytase XP 897420; Garant Tiernahrung GmbH, Pöchlarn, Austria). In both diets, the Ca/tP ratio was adjusted to 1.3 to 1, whereas the Ca/aP ratio corresponded to 2.5 to 1 and 2.0 to 1 for the Con and Phy diets, respectively [12,13]. Pigs had free access to pelleted diets via transponder-based feeders (feed intake recording equipment feeder; Schauer Agrotonic GmbH, Prambachkirchen, Austria). By weighing the trough before and after the individual pig had access to the feeder in the pen, the consumed amount of feed was automatically recorded (Farm Manager, Schauer Agrotonic GmbH, Austria) and the weights of the individual meals separately for each pig were collated daily. Feed samples from each dietary group were collected on experimental days 1, 22, and 49. The performance parameters of all pigs were calculated and published in Vötterl et al [5]. Here, performance data of the 24 pigs selected for sampling are presented. The health status of the pigs was monitored daily by visual inspection and by checking their individual feed intake.

### Intestinal sampling

Intestinal samples were collected from a subset of 24 pigs (n = 12/diet; 6 gilts and 6 barrows) on experimental days 50 and 51. We collected the samples in the morning of two consecutive days to keep the time frame of sampling narrow to account for diurnal variation in hormonal secretions. On each sampling day, three gilts and three barrows, one per pen, with an average BW were selected. The pigs were stunned by the captive bolt and subsequently exsanguinated by opening both jugular veins. Blood samples were collected from the opened jugular veins and stored on ice providing the same conditions throughout replicate batches before being centrifuged at 2,700×g for 10 min at 4°C (Eppendorf Centrifuge 5804 R; Eppendorf, Hamburg, Germany). The collected serum was stored at −20°C. For gene expression analysis, the duodenum (20 cm caudal from pylorus sphincter), mid-jejunum (30 cm), ileum (20 cm cranial from ileocecal sphincter), whole cecum, and mid-colon (30 cm) were separated, opened at the mesenterium, emptied of digesta, which was sampled, placed on ice and further stored at −20°C. The emptied gut pieces were washed in ice-cold phosphate-buffered saline and dry-blotted with paper towels. The mucosa of each segment was scraped off with a microscopic glass slide and snap-frozen in liquid nitrogen before being stored at −80°C. From the right kidney, a subsample at the margo lateralis of the cortex was taken and treated like the intestinal mucosal scrapings. Right metacarpal bones were collected, weight and length were recorded, then placed on ice and further stored at −20°C. From death, collection and first processing of samples took less than 27±7 min.

### Diet analyses, serum and bone parameters, and short-chain fatty acid analysis

Chemical analysis of dry matter (DM), crude ash, crude protein, crude fat, Ca, P, total starch, and non-resistant starch in feed samples were conducted as previously described in Vötterl et al [5]. The content of aP was calculated using data for aP contents in individual feed ingredients either without or with phytase addition reducing inorganic P supply by 1 g per diet [12]. LUFA Nord-West analyzed phytase activity after the DIN EN ISO 30024 method (Institut für Futtermittel; LUFA Nord-West, Oldenburg, Germany).

The serum content of phosphorus, calcium, alkaline phosphatase, cholesterol, non-esterified fatty acids, triglycerides, and urea were analyzed using enzymatic colorimetric assays on an autoanalyzer for clinical chemistry (Cobas 6000/c501; Roche Diagnostics GmbH, Basel, Switzerland). Enzyme-linked immunosorbent assays (ELISA) were used to determine fibroblast growth factor (FGF23; Porcine FGF23 ELISA Kit [EP0058], Fine Test, Wuhan Fine Biotech Co., Ltd., Hubei, China; coefficient of variation [CV] <10%), 25-hydroxyvitamin D3 and D2 (25-Hydroxy Vitamin D EIA [AC-57SF1], Immunodiagnostic Systems Holdings PLC, Boldon, United Kingdom; CV<20%) and osteocalcin (N-MID Osteocalcin ELISA [AC-11F1], Immunodiagnostic Systems Holdings PLC, Boldon, United Kingdom; CV<6%).

The third metacarpal bone was cut into two halves in the middle of the diaphysis shaft. Then, the external (H) and internal (h) horizontal diameters from the medial to lateral side of the bone as well as the external (B) and internal (b) vertical diameters from the dorsal to plantar side of the bone were measured with a caliper. These diameters were used for the calculation of the geometrical properties cortical wall thickness ([(B+H)–(b+h)]/4), cortical wall area (π/4 ×[(B×H) −(b×h)]), and cortical index ([(H–h)/H]+[(B–b)/B]) to estimate the relative ratio of the cortical wall to the total width of the bone [1,14].

The SCFAs including acetic, propionic, isobutyric, butyric, isovaleric, valeric, caproic, and heptanoic acid in the digesta of the stomach, cecum, and mid colon were determined using gas chromatography (GC) according to a previously described protocol [14]. The mid jejunum and ileum of more than half of the pigs were empty during sample collections. Therefore, the SCFA in jejunal and ileal digesta were not determined. Briefly, 1.0 g of digesta sample was mixed with 200 μL of 25% phosphoric acid and 300 μL of internal standard (4-methylvaleric acid; Sigma-Aldrich, St. Louis, MO, USA). To the gastric digesta approximately 500 μL, and to the cecal and colonic digesta 1,000 μL of double-distilled water were added. Samples were centrifuged (20,000×g for 20 min) to use the clear supernatant for measuring the SCFA on the GC-2010 Plus Capillary GC (Shimadzu Corp., Kyoto, Japan) using a 30 m×0.53 mm×0.5 μm capillary column (Trace TR Wax; Thermo Fisher Scientific, Waltham, MA, USA) and helium as a carrier gas. The gas chromatograph was equipped with an autosampler and injector (AOC-20s Auto Sampler; AOC-20i Auto-Injector; Shimadzu Corp., Kyoto, Japan) and a flame-ionization detector (BID-2010 Plus; Shimadzu Corp., Japan).

### Gene expression

In total, 14 target genes in intestinal mucosal samples (vitamin D receptor [*VDR*]; cytochrome P450, family 24, subfamily A, polypeptide 1 [*CYP24A1*]; transient receptor potential vanilloid 5 [*TRPV5*]; transient receptor potential vanilloid 6 [*TRPV6*]; calbindin 1 [*CALB1*]; plasma membrane Ca^2+^ adenosine triphosphatase 1b [*PMCA1b*]; *FGF23*; Na+-Pi cotransporter 1 [*SLC34A1*]; Na+-Pi cotransporter 2 [*SLC34A2*]; Na+-Pi cotransporter 3 [*SLC34A3*]; claudin-4 [*CLDN4*]; occluding [*OCLN*]; zonula occludens-1 [*ZO1*]; cadherin-1 [*CDH1*]) and 9 target genes in renal samples (*CYP24A1*, *TRPV5*, *TRPV6*, *CALB1*, *PMCA1b*, *FGF23*, *SLC24A1*, *SLC34A2*, *SLC34A3*) related to the Ca and P homeostasis were amplified together with five reference genes (β-actin [*ACTG*]; glyceraldehyde-3-phosphate-dehydrogenase [*GAPDH*]; β2-microglobulin [*B2M*]; hypoxanthine-guanine phosphoribosyl transferase [*HPRT*]; ornithine decarboxylase antizyme [*OAZ1*]). All samples were analyzed with two technical replicates per sample. From 20 mg of each intestinal mucosa (duodenum, jejunum, ileum, cecum, and colon) and kidney sample, total RNA was isolated using mechanical homogenization and the RNeasy Mini Kit (RNeasy Mini Qiacube Kit; Qiagen, Hilden, Germany), treatment with DNase I (Invitrogen TURBO DNA-free Kit; Thermo Fisher Scientific Inc., Waltham, MA, USA). RNA was evaluated on the Qubit Fluorometer using Qubit RNA HS Assay for quantification and the Qubit RNA IQ Assay (Qubit 4 Fluorometer; Qubit RNA HS Assay Kit; Qubit RNA IQ Assay, Thermo Fisher Scientific Inc., USA) for quality control [14]. If the RNA integrity number was below eight, the RNA was newly isolated from the respective sample. Complementary DNA (cDNA) was synthesized using the High Capacity cDNA RT Kit following the manufacturer’s protocol (High-Capacity cDNA Reverse Transcription Kits; Thermo Fisher Scientific Inc., USA). Amplification and quantification of the cDNA were performed with Fast-Plus EvaGreen Master Mix with Low ROX (Biotium, Fremont, CA, USA) on a ViiA 7 Real-Time PCR System (Thermo Fisher Scientific, USA). The pipetting for quantitative polymerase chain reaction (qPCR) was conducted using a robot (epMOTION 5057TMX; Eppendorf AG, Germany). Melting curve analysis was performed to verify the specificity of the PCR amplification. Standard curves were generated using serial dilutions (10^−3^ to 10^−7^ molecules/μL) of the purified products (QIAquick PCR Purification Kit; Qiagen, Germany) and quantified PCR products (Qubit dsDNA HS Assay Kit; Thermo Fisher Scientific, USA) generated by qPCR from the samples. Each standard and sample reaction contained master mix (7 μL) including 5 μL of Fast-Plus EvaGreen MasterMix with Low ROX, 0.2 μL forward and reverse primers, and 25 ng of cDNA. After an initial denaturation step at 95°C for 10 min, 40 cycles of 95°C for 10 s followed, primer annealing at 60°C for 30 s, and elongation at 72°C for 30 s. Throughout all cycles, fluorescence was measured. Beforehand, primers for amplification of target and housekeeping genes were checked for accuracy [14] or newly designed using PrimerBLAST ( www.ncbi.nlm.nih.gov/tools/primer-blast/). The amplification efficiencies for all primers (E = 10(−1/slope)) are provided in the Supplementary Table S1. Gene expression was calculated as transcript copies per gram sample (QM×C×DV)/(S×W) with QM, quantitative mean copy number; C, cDNA concentration of each sample, DC, dilution volume of cDNA; S, cDNA amount [ng]; W, weight of cDNA sample [g] subjected to qPCR) [15].

### Statistical analysis

After testing the data for normal distribution using the Shapiro–Wilk test in SAS (version 9.4, SAS Inst. Inc., Cary NC, USA), data from the 24 selected pigs were subjected to analysis of variance using the MIXED procedure. The model accounted for the fixed effects of phytase, sex, and the interaction phytase×sex. Random measures were used to assess differences between gut segments. Replicate batch was considered as random effect and the individual pig nested within litter (pen) as the experimental unit assuming a compound symmetry variance-covariance structure (type = cs). Degrees of freedom were approximated by the Kenward-Roger method. Data were expressed as least squares means±standard error of the mean (SEM). Significance was defined at p≤0.05 and trends were discussed at 0.05<p≤0.10. The pairwise comparisons between least-square means were performed using the PDIFF option in SAS. Pearson correlation coefficients were calculated between mineral intake, serum parameters, and bone parameters and between SCFA concentration and gene expression of tight and adherens-junction proteins in the respective gut site using PROC CORR of SAS. A significant correlation was defined as p≤0.05 and |r|≥0.35. In addition, the canonical correlation analysis was conducted with the same data set as for the Pearson correlation analysis using PROC CANCORR of SAS.

## RESULTS

### Diet and animal performance

The majority of pigs were clinically healthy throughout the study. Irrespective of dietary treatment, ten pigs were removed from the experiment in the first four weeks (replicate batch 1: 7 pigs; replicate batch 2: 3 pigs) due to tail biting or low feed intake. The analyzed phytase activities were 257 and 596 FTU/kg complete diet for Con and Phy diets, respectively, resulting in a 24.9% higher aP content in Phy diet compared to Con diet (Table 1). In the subset of 24 pigs, average daily feed intake (ADFI) was 13.2% higher in barrows (Table 2), leading to a 13.2%, 13.1%, and 13.2% higher tP, aP, and Ca intake in barrows compared to gilts, respectively (p = 0.001). Phytase supplementation reduced the pig’s ADFI and ADG by 8.7% and 9.2% compared to Con diet (p<0.05). This caused a lower tP and Ca intake by 10.1% and 8.4%, respectively, whereas the aP intake increased by 14.1% (p<0.001) in pigs fed Phy diet compared to those fed Con diet (p<0.05). The performance data from all pigs are in the Supplementary Table S2.

### Serum and bone parameters and short-chain fatty acids

In the subset of 24 pigs serum parameters were not altered by phytase supplementation, except for the trend for a phytase ×sex interaction (p = 0.058) for FGF23 (Table 3). By contrast, phytase supplementation decreased (p = 0.035) the cortical index of metacarpal bone by 7.7% due to its decreasing effect (trend, p = 0.066) on the cortical wall thickness of metacarpal bones compared to Con diet (Table 4). Phytase supplementation reduced levels of gastric propionate, cecal valerate, and colonic caproate by 58.3%, 38.6%, and 46.7%, respectively (Table 5; p<0.05). The serum results for all 144 fattening pigs are in the Supplementary Table S3.

### Gene expression along the intestinal tract and in the kidney

Phytase supplementation increased the expression of Ca transport and binding proteins *TRPV5*, *TRPV6*, *CALB1*, and *PMCA1b* in the duodenum compared to the Con diet (p<0.05; Table 6). Furthermore, phytase supplementation increased the colonic *FGF23* expression by 6.9% and cecal *SLC34A2* expression by 6.8% compared to Con diet (p<0.05). The phytase×sex interaction for the expression of *TRPV5* indicated that barrows fed the Con diet expressed less TRPV5 in the colon compared to all other groups (p = 0.014). Additionally, phytase supplementation increased the expression of tight and adherens-junction proteins *CLDN4*, *OCLN*, *ZO1*, and *CDH1* by 13.2% in the duodenum and of *CLDN4* by 4.7% in the colon compared to the Con diet (p<0.05; Table 7). Barrows had 4.1% and 4.2% lower colonic expression of the tight-junction protein *CLDN4* and adherens-junction protein *CDH1* (p = 0.043), respectively, compared to gilts (Table 7). Renal gene expression was similar between diets (Table 8). Sex-related differences existed, showing that barrows expressed less *TRPV5* and *SLC34A3* in the kidney compared to gilts (p<0.05).

### Pearson’s correlation between mineral intake, serum, and bone parameters and between short-chain fatty acid concentration and gene expression of tight- and adherens junction proteins

Daily tP intake correlated negatively with metacarpal bone weight (r = −0.47) and metacarpal bone cortical wall thickness (r = −0.45), but it positively correlated with metacarpal bone cross-sectional area (r = 0.88) and cortical index (r = 0.68; Table 9). In addition, average daily Ca intake positively correlated with aP intake (r = 0.44). In serum, P correlated negatively with FGF23 (r = −0.65) and the Ca/P ratio (r = −0.89; Table 9). Likewise in serum, Ca/P ratio correlated positively with FGF23 (r = 0.49) and Ca with alkaline phosphatase (r = 0.44). Furthermore, serum VitD correlated positively with metacarpal bone length (r = 0.59). Metacarpal bone weight correlated positively with metacarpal bone cortical wall thickness (r = 0.89), whereas the metacarpal bone cortical index was positively associated with its cross-sectional area (r = 0.95; Table 9). In the stomach, valerate correlated negatively with the expression of *CLDN4*, *OCLN*, and *ZO1* in the duodenum (r<−0.44, Supplementary Table S4). Moreover, valerate and heptanoate correlated negatively with the expression of *CLDN4* in the cecum (r<−0.46).

### Canonical correlation between mineral intake, serum, and bone parameters and between short-chain fatty acid concentration and gene expression of tight- and adherens junction proteins

**Figure 1:**
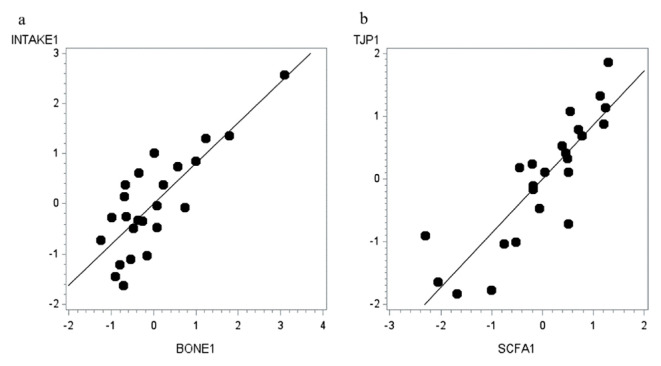
Scatter plots of the first canonical function correlating (a) mineral intake with bone parameters (p = 0.033); and (b) short-chain fatty acids with relative gene expression of tight- and adherens-junction proteins in the colon (p = 0.030).

The first canonical function of the canonical correlation analysis (CCA) showed a positive correlation between mineral intake and bone parameters (canonical correlation = 0.812; p = 0.033; Figure 1). This was based on the correlation of Ca intake with the first canonical variables of bone parameters (canonical coefficient = 0.63), as well as on the correlations of cortical wall thickness (canonical coefficient = 0.32) and of the cross-sectional area (canonical coefficient = 0.39) with the first canonical variables for mineral intake as these represented the highest correlation for the first canonical function. Similarly, the first canonical function of the CCA showed a positive correlation between SCFA and relative expression of tight- and adherens junction proteins in colon (canonical correlation = 0.862; p = 0.030; Figure 2). Additionally, the negative correlation of the relative expression of *CDH1* (canonical coefficient = −0.65) and *CLDN4* (canonical coefficient = −0.39) with the first canonical variables of SCFA parameters contributed to the first canonical function. Likewise, the negative correlation of acetate (canonical coefficient = −0.65) and heptanoate (canonical coefficient = −0.64) with the first canonical variables of the relative expression levels of tight- and adherens junction proteins supported the significant first canonical correlation.

## DISCUSSION

In this study, we investigated the long-term effects of two dietary aP levels via supplementation of phytase on the Ca and P homeostasis, specifically on serum and bone parameters as well as on the related gene expression in the intestine and kidney in growing gilts and barrows. The analysis of phytase activity suggested an increase in dietary aP of almost 25%, presumably increasing the total tract digestibility of P as previously shown [14,16]. As the phytase-induced elevated aP levels were fed continuously for eight weeks, it can be assumed that certain phytase effects, including those related to intestinal absorption, renal excretion, and regulatory hormones in serum and bone parameters, were the result of a long-term adaptation to increased dietary aP provision. In this regard, it should be also considered that we used a one-phase feeding system to simulate the conditions on small-scale farms in which nutrients are generally at the lower range to meet their daily requirements at the beginning of the growing phase, whereas they are at a surplus in the late finishing phase. The lack of sex×phytase interaction for most parameters in our study implied that phytase effects were similarly directed and mediated in both sexes. We selected the pigs for the invasive sampling based on equal growth performance. However, it is here important to note that the barrows still consumed more feed and weighed more than the gilts, which led to regulatory adaptations, for instance in the colon for Ca absorption and in the kidney for Ca and P (re-)absorption. Of note, the phytase supplementation reduced the ADFI while the expression of genes related to Ca uptake in the duodenum was upregulated, potentially as a measure to balance the improved P absorption. Regulatory action due to the phytase-induced changed Ca and P availability by the kidneys was not detectable at the gene expression level, but bone metrics pointed towards an imbalance in the systemic availability of Ca and P for bone formation.

The present study was conducted at our university pig farm and the diets were mixed at a commercial feed mill. Consequently, the experimental conditions were similar to commercial pig fattening and results are therefore relevant to the general practice. This comprised the dietary supplementation of phytase and losses observed during diet manufacturing (due to pelleting) which remained in the tolerable range as the dietary phytase activity was above 500 FTU/kg complete feed. As storage time, humidity, and temperature can negatively affect phytase activity [17], we took precautions by controlling our storage conditions in order not to further reduce of the selected phytase activity.

Due to the higher intestinal availability of P with the Phy diet, a down-regulation of P uptake in the small intestine, the major absorption site of P, and up-regulation of renal excretion as a long-term regulation could have been assumed. Instead, we found similar expression levels of sodium-phosphate co-transporters in the intestines and kidney (*SLC34A1* being expressed highest in the kidney) between dietary groups, which would support increased retention of P. As there was almost no regulation in intestinal and renal P transporter expression, the addition of phytase might have counterbalanced the 10%-reduction in ADFI and subsequently lowered Ca and tP intake with the Phy diet. It might be possible that smaller or less frequent meals throughout the day with the Phy diet increased gastric acidification, which, in turn, may have optimized the activity of the intrinsic and added phytase. Greater duodenal expression of *TRPV5*, *TRPV6*, *CALB1*, and *PMCA1b*, covering all components of the three-step process in the epithelial Ca transport, with Phy diet would support an increased intestinal Ca absorption to balance the intestinal P uptake and compensate for the lower Ca intake with the Phy diet due to lowered ADFI. Under the practical housing conditions, it was not possible to perform a balance study; therefore, valuable data for the regulation of Ca and P excretion via kidneys and intestines could not be gained. In addition, gene expression data provide a first indication but do not reflect the actual amount of functional protein; consequently, no definite conclusion about the involvement especially of the kidneys can be drawn. The aforementioned intestinal and renal adjustments may partly explain the missing effect of phytase supplementation on serum levels of Ca and P as well as regulatory hormones after eight weeks on the diet. However, the fact that pigs fed the Phy diet had a lower cortical index of metacarpal bone would support that their body tried to compensate for lower ADFI and mineral intake and hence modulating intestinal and renal (re-)absorption of minerals to maintain bone structure without being apparent on gene expression level. We did not measure mineral density or breaking strength in the present study [18,19]. Therefore, the effect of phytase supplementation on overall bone strength remains speculative.

Feed intake inhibition and growth depression have been reported for high intakes of Ca, also in weaned pigs, but not specifically for P [20,21]. The negative effect of Ca on BW has been linked to changes in appetite regulation, nutrient assimilation, and energy metabolism [20,21]. However, in these studies intestinal Ca and P availabilities were nutritionally modified together and, as such, need to be interpreted together. Therefore, similar modes of action may be feasible for the present reduced ADFI and ADG with the phytase addition. Nevertheless, the feed efficiency was not affected, an important criterion in pig production. Under the practical conditions, in which we conducted the study, total collection of urine was hard to achieve, not allowing us to calculate Ca and P balance which would have helped to better understand the present observations for growth performance. Hence, high P intake and improved P digestibility through phytase supplementation may be less effective in improving performance in well-growing pigs at the end of their fattening period [22].

The 24 piglets were selected on the basis that they represented average BW and ADG per pen and dietary group. Nevertheless, the barrows in this subset of 24 selected pigs still had higher ADG compared to gilts, which could be related to their higher ADFI [23]. The latter may have been the underlying reason for sex-related differences observed in serum and intestinal gene expression, especially for the diverging results for barrows fed the Con diet compared to the other pig groups. With the major action of FGF23 being the suppression of phosphate reabsorption [24], the trend for lower serum FGF23 in barrows fed the Con diet, which had the highest ADFI, may support increased P utilization for bone growth. In the same group of barrows fed the Con diet, the trends for higher *FGF23* expression in the duodenum and lower expression of *TRPV5*, *PMCA1b*, and tight- and adherens-junction proteins in the colon further suggested a lower intestinal trans- and paracellular Ca and P uptake to adjust the higher dietary intake and balance systemic availability of Ca and P. Although the intestinal Ca absorption is VitD dependent, present data did not show the respective associations for intestinal and renal expression of the *VDR* and *CYP24A1* or for serum VitD. However, diets contained adequate amounts of VitD and pigs were in different stages of digestion and post-absorptive metabolism, potentially masking a phytase effect. Moreover, translational adjustments in the VDR protein abundance across groups cannot be ruled out. Aside from a greater duodenal transcellular absorption, phytase-related effects seemed to modulate the paracellular absorption of Ca. The duodenal expression of tight-junction proteins *CLDN4*, *OCLN* and *ZO1* that form size- and charge-selective pores as well as of adherens-junction protein *CDH1* which is important for tight junction formation were greater with the Phy diet [25,26] may have indicated an increased paracellular Ca absorption. Information about further pore-forming claudin–members like claudin-2 and -12, which we did not include in the set of genes in this study, would be relevant genes to investigate in the future to support our assumption [27]. Besides, tight- and adherens-junction proteins are crucial components of the physical intestinal barrier, responding to alterations in exposure to luminal antigens of dietary or microbial nature [28]. The significant first canonical function between SCFA and tight- and adherens-junction proteins would confirm this assumption. The impact of the high abundant acetate as well as low abundant heptanoate on this canonical correlation demonstrate that each shift in SCFA profile may influence barrier function. Therefore, it seems possible that some phytase-related effects were mediated via changes in the microbial community or fermentation. Phytase supplementation caused shifts in all major bacterial taxa and reduced species richness and α-diversity in the feces of the present pigs [11]. The decreased levels of gastric propionate, butyrate, and valerate with phytase supplementation may indicate reduced microbial metabolic activity. However, this may have been also the result of the lower ADFI, providing less fermentable substrate or a misbalance in the microbial availability of P to Ca as they are crucial for microbial metabolism. In support of this assumption, single SCFA, most of all propionate, decreased with phytase feeding but not total SCFA, suggesting that specific bacterial groups or enzymes were inhibited. Furthermore, SCFAs can contribute to lowering gastric pH (i.e. in the corpus and pylorus region) depending on their pKa value, stomach filling, and gastric retention time that influences digestive and fermentative processes. Like in gastric digesta, less valerate and caproate in the cecum and colon may be the result of the lower microbial substrate and mineral availability due to the reduced ADFI and changes in the intestinal Ca and P availability in pigs fed the Phy diet. Without having measured the pH in digesta, we can only guess whether this shift in SCFA proportions further affected the Ca and P solubility and consequently their absorption [14,29].

Assumable from the results of all pigs used in this research [5], linking the daily intake of Ca, tP, and aP with serum parameters proved to be difficult in the subset of 24 pigs at the time point of sampling. Nevertheless, the Pearson correlation analysis suggested additional markers for dietary P intake as indicated by the weak to strong correlations between the tP intake and metacarpal bone cortical index and cross-sectional area. Likewise, the Canonical correlation analysis demonstrated an association between Ca intake and cortical wall thickness and cross-sectional area. This highlights the importance of an adequate Ca and P intake for bone structure as well as the complex interplay of Ca and P in bone metabolism. Despite being invasive as it involves removing animals from a pig group, these bone parameters may allow a good estimation of the adequacy of P and Ca provision during the fattening period. If no skeletal problems occurred, it may be also thinkable to evaluate the dietary adequacy retrospectively, assessing the bones after slaughtering the pigs, to receive this information for the next batch of pigs.

In conclusion, results showed that supplementation of pig’s diet with phytase under practical conditions and free access to the diets for eight weeks resulted in a lower ADFI, causing lower Ca and tP intake and consequently depressed ADG but did not affect feed conversion ratio. Despite the lower tP intake, phytase supplementation increased the aP intake, which did not modify the intestinal expression of regulatory elements for P absorption. Instead, intestinal gene expression suggested elevated duodenal trans- and paracellular Ca absorption which seemed to balance the increased P uptake. Similar serum levels of Ca and P and regulatory hormones supported this assumption. Furthermore, the phytase-related shifts in the SCFA profile may have been related to the differences in intestinal Ca and P availability or to the reduced ADFI and thus microbial substrate. The altered microbial activity may have had consequences for the intestinal absorption of Ca. Observed effects of sex on intestinal and renal expression of genes related to Ca and P homeostasis were likely caused by the lower ADFI in gilts than in barrows.

## Figures and Tables

**Table 1:** Ingredients and chemical composition of the experimental diets

Items	Control diet	Phytase diet
Ingredients (%)		
Barley	45.06	45.00
Wheat (11% CP)	35.51	35.46
Soybean meal HP (47% CP)	8.11	8.10
Soybean meal (42% CP)	7.01	7.00
Calcium carbonate	1.28	1.28
Rapeseed oil	1.00	1.00
Monocalcium phosphate	0.46	0.46
Salt	0.46	0.46
Lysine-HCL 98	0.41	0.41
Premix^1),2)^	0.39	0.39
L-Threonine	0.13	0.13
Magnesium oxide	0.10	0.10
DL-Methionine	0.08	0.08
Phytase (650 phytase units/kg)	-	0.13
Chemical composition (%)		
Dry matter	89.3	89.3
Crude ash	5.23	5.31
Crude protein	18.5	18.4
Crude fiber	3.80	4.00
Crude fat	3.70	3.70
Nitrogen-free extract	69.2	69.1
Total starch	50.4	51.1
Resistant starch	0.34	0.34
Non-resistant starch	49.0	48.9
Calcium	7.48	7.50
Phosphorus	5.68	5.59
Available phosphorus	2.95	3.68
Phytase^3)^ (phytase units/kg diet)	257	596
Metabolizable energy (MJ/kg diet)	15.2	15.1

1) The vitamin-mineral-premix without phytase provided per kilogram of experimental diet (Garant GmbH, Pöchlarn, Austria): 6,510 IE vitamin A, 2,003 IE vitamin D_3_, 104.973 mg vitamin E, 3.005 mg vitamin K_3_, 1.502 mg vitamin B_1_, 4.006 mg vitamin B_2_, 20.031 mg vitamin B_3_, 2.003 mg vitamin B_6_, 0.020 mg vitamin B_12_, 10.016 mg Pantothenic acid, 0.501 mg folic acid, 0.050 mg biotin, 1,242.038 mg choline, 132.172 mg choline chloride, 160.460 mg Fe, 21.567 mg Cu, 122.060 mg Zn, 67.358 mg Mn, 0.860 mg Mo, 1.721 mg J, 0.102 mg Co, 0.568 mg Se.

2) The vitamin-mineral-premix with phytase provided per kilogram of experimental diet (Garant GmbH, Pöchlarn, Austria): 6,502 IE vitamin A, 2,001 IE vitamin D_3_, 104.837 mg vitamin E, 3.001 mg vitamin K_3_, 1.500 mg vitamin B_1_, 4.001 mg vitamin B_2_, 20.005 mg vitamin B_3_, 2.001 mg vitamin B_6_, 0.020 mg vitamin B_12_, 10.003 mg pantothenic acid, 0.500 mg folic acid, 0.050 mg biotin, 1,240.434 mg choline, 132.000 mg choline chloride, 160.251 mg Fe, 21.539 mg Cu, 121.901 mg Zn, 67.270 mg Mn, 0.859 mg Mo, 1.719 mg J, 0.102 mg Co, 0.567 mg Se.

3) 6-Phytase (VM Phytase XP 897420, Garant-Tiernahrung GmbH, Pöchlarn, Austria).

**Table 2:** Effect of dietary phytase and sex on body weight, average daily feed intake, calcium intake, total phosphorus intake, available phosphorus intake, average daily gain, and feed conversion ratio in fattening pigs between experimental days 1 and 49 (n = 12 per diet; 6 gilts and 6 barrows)

Items	Control diet		Phytase diet		SEM	p-values		
		
	Gilts	Barrows	Gilts	Barrows		Phytase	Sex	Phytase×sex
Body weight day 1 (kg)	39.0	38.3	40.5	39.7	1.92	0.464	0.694	1.000
Body weight day 49 (kg)	84.4	86.7	81.1	84.3	2.11	0.192	0.207	0.845
Average daily feed intake (kg/d)	2.07	2.35	1.89	2.14	0.070	0.012	0.001	0.832
Calcium intake (g/d)	17.2	19.5	15.8	17.9	0.58	0.015	0.001	0.841
Total phosphorus intake (g/d)	13.1	14.8	11.8	13.3	0.44	0.004	0.001	0.816
Available phosphorus intake (g/d)	6.81	7.71	7.77	8.79	0.252	0.001	0.001	0.830
Average daily gain (kg/d)	0.95	1.01	0.85	0.93	0.037	0.026	0.058	0.825
Feed conversion ratio	2.20	2.32	2.27	2.32	0.068	0.628	0.234	0.628

SEM, standard error of the means.

**Table 3:** Effect of dietary phytase and sex on serum parameters related to the calcium and phosphorous homeostasis in fattening pigs sampling on experimental days 50 and 51 (n = 12 per diet; 6 gilts and 6 barrows)

Items	Control diet		Phytase diet		SEM	p-values		
		
	Gilts	Barrows	Gilts	Barrows		Phytase	Sex	Phytase×sex
Phosphorus (mmol/L)	3.04	2.95	3.09	2.78	0.136	0.664	0.158	0.443
Calcium (mmol/L)	2.65	2.62	2.63	2.64	0.04	0.985	0.837	0.723
Calcium/phosphorus ratio	0.88	0.90	0.87	0.95	0.04	0.562	0.171	0.381
Fibroblast growth factor 23 (ng/L)	814	601	704	903	102.4	0.360	0.949	0.058
Vitamin D (U/L)	17.4	16.6	19.8	17.3	1.44	0.299	0.261	0.552
Alkaline phosphatase (U/L)	147	142	161	149	14.6	0.509	0.575	0.822
Osteocalcin (μg/L)	23.3	26.5	27.9	29.6	2.72	0.174	0.377	0.779
Urea (mmol/L)	7.26	7.94	8.45	9.06	1.005	0.265	0.530	0.972
Cholesterol (mmol/L)	2.28	2.61	2.37	2.32	0.183	0.605	0.457	0.323
Triglyceride (mmol/L)	0.35	0.41	0.44	0.33	0.050	0.869	0.668	0.117
Non-esterified fatty acids (mmol/L)	3.04	2.95	3.09	2.78	0.136	0.664	0.158	0.443

SEM, standard error of the means.

**Table 4:** Effect of dietary phytase and sex on metacarpal bone parameters in fattening pigs on experimental days 50 and 51 (n = 12 per diet; 6 gilts and 6 barrows)

Items	Control diet		Phytase diet		SEM	p-values		
		
	Gilts	Barrows	Gilts	Barrows		Phytase	Sex	Phytase×sex
Morphometric parameters								
Length (cm)	7.08	6.83	7.00	7.58	0.266	0.226	0.539	0.134
Weight (g)	55.9	53.6	55.3	64.7	4.84	0.288	0.475	0.239
Geometrical parameters								
Cortical wall thickness (mm)	1.96	1.98	1.73	1.84	0.09	0.066	0.527	0.658
Cross-sectional area (mm^2^)	72.5	70.9	61.4	68.4	4.57	0.152	0.561	0.362
Cortical index	0.56	0.59	0.53	0.54	0.02	0.035	0.361	0.577

SEM, standard error of the means.

**Table 5:** Effect of dietary phytase and sex on short-chain fatty acid concentrations in gastric, cecal, and colonic digesta of fattening pigs on experimental days 50 and 51 (n = 12 per diet; 6 gilts and 6 barrows)

Items	Control diet		Phytase diet		SEM	p-values		
		
	Gilts	Barrows	Gilts	Barrows		Phytase	Sex	Phytase×sex
Gastric digesta (μmol/g)								
Acetate	4.35	3.98	4.05	7.42	2.647	0.561	0.578	0.489
Propionate	0.37	0.43	0.18	0.15	0.068	0.003	0.810	0.472
Isobutyrate	0.35	0.47	0.50	0.38	0.087	0.705	1.000	0.194
Butyrate	0.12	0.10	0.02	0.07	0.037	0.087	0.656	0.377
Isovalerate	ND	ND	ND	ND	-	-	-	-
Valerate	0.03	0.03	0	0	0.019	0.091	1.000	1.000
Caproate	0.02	0	0	0.02	0.011	1.000	1.000	0.163
Heptanoate	ND	ND	ND	ND	-	-	-	-
Total amount	5.23	5.02	4.77	8.02	2.723	0.647	0.584	0.532
Cecal digesta (μmol/g)								
Acetate	95.30	96.52	108.85	104.67	7.788	0.180	0.851	0.733
Propionate	38.02	42.40	37.08	39.65	5.568	0.745	0.540	0.872
Isobutyrate	1.40	1.20	1.57	1.77	0.249	0.158	1.000	0.432
Butyrate	15.63	13.08	11.12	10.00	2.457	0.139	0.465	0.774
Isovalerate	1.73	1.45	1.90	2.17	0.321	0.184	0.980	0.402
Valerate	3.08	4.38	2.08	2.50	0.626	0.033	0.186	0.489
Caproate	0.33	0.33	0.07	0.28	0.079	0.059	0.185	0.185
Heptanoate	0.17	0.32	0.07	0.15	0.049	0.077	0.143	0.867
Total amount	155.68	159.57	162.77	161.18	14.199	0.763	0.936	0.849
Colonic digesta (μmol/g)								
Acetate	127.20	102.90	114.20	120.75	11.494	0.835	0.450	0.195
Propionate	45.68	38.67	34.53	37.40	4.015	0.111	0.529	0.191
Isobutyrate	3.35	3.83	3.73	4.10	0.469	0.497	0.376	0.902
Butyrate	22.32	19.30	16.82	17.52	2.555	0.170	0.655	0.476
Isovalerate	4.45	5.05	4.73	5.38	0.718	0.672	0.395	0.973
Valerate	4.93	4.30	3.43	3.72	0.559	0.078	0.758	0.422
Caproate	0.75	0.75	0.40	0.40	0.156	0.037	1.000	1.000
Heptanoate	0.30	0.32	0.30	0.30	0.046	0.857	0.857	0.857
Total amount	210.00	175.13	178.10	189.58	18.578	0.644	0.537	0.227

SEM, standard error of the means; ND, not detectable.

**Table 6:** Effect of dietary phytase on gene expression related to mineral absorption along the intestinal tract of fattening pigs (log_10_ copies cDNA/g sample) on experimental days 50 and 51 (n = 12 per diet; 6 gilts and 6 barrows)

Gene^1)^	Gut site^2)^	Control diet		Phytase diet		SEM	p-values		
		
		Gilts	Barrows	Gilts	Barrows		Phytase	Sex	Phytase×sex
*VDR*	Duodenum	6.8	6.7	6.8	7.2	0.21	0.157	0.449	0.292
	Jejunum	6.7	7.0	7.0	7.6	0.30	0.176	0.126	0.536
	Ileum	ND	ND	ND	ND	-	-	-	-
	Cecum	6.6	7.1	6.5	6.8	0.28	0.520	0.222	0.919
	Colon	6.9	6.8	7.2	7.0	0.20	0.292	0.453	0.714
*CYP24A1*	Duodenum	6.1	6.3	6.6	6.9	0.43	0.165	0.585	0.923
	Jejunum	ND	ND	ND	ND	-	-	-	-
	Ileum	ND	ND	ND	ND	-	-	-	-
	Cecum	7.3	8.0	7.2	6.6	0.55	0.211	0.898	0.249
	Colon	7.5	7.4	6.6	6.9	0.62	0.292	0.906	0.745
*TRPV5*	Duodenum	7.6	7.8	8.9	8.5	0.34	0.011	0.806	0.398
	Jejunum	7.4	7.3	7.4	7.9	0.34	0.385	0.668	0.370
	Ileum	6.9	6.8	6.3	7.3	0.47	0.981	0.363	0.256
	Cecum	7.9	7.4	7.6	7.7	0.25	0.971	0.483	0.323
	Colon	7.8^a^	6.8^b^	7.4^a^	7.6^a^	0.22	0.388	0.088	0.014
*TRPV6*	Duodenum	7.8	8.1	8.8	8.7	0.37	0.049	0.809	0.645
	Jejunum	ND	ND	ND	ND	-	-	-	-
	Ileum	ND	ND	ND	ND	-	-	-	-
	Cecum	ND	ND	ND	ND	-	-	-	-
	Colon	7.6	7.0	7.3	7.6	0.39	0.701	0.749	0.304
*CALB1*	Duodenum	8.4	9.0	9.7	9.7	0.38	0.018	0.442	0.387
	Jejunum	7.8	7.8	7.6	8.3	0.40	0.786	0.400	0.416
	Ileum	6.8	6.2	5.8	6.0	0.52	0.275	0.705	0.380
	Cecum	6.1	6.7	6.4	6.1	0.30	0.655	0.647	0.208
	Colon	6.7	6.3	6.6	6.5	0.17	0.712	0.189	0.506
*PMCA1b*	Duodenum	8.3	8.9	10.1	10.1	0.60	0.023	0.641	0.663
	Jejunum	8.6	8.4	8.4	9.7	0.53	0.352	0.304	0.197
	Ileum	7.5	6.8	6.7	7.1	0.80	0.707	0.867	0.518
	Cecum	9.4	9.7	9.9	9.9	0.44	0.392	0.796	0.667
	Colon	10.3	9.3	10.1	10.2	0.23	0.335	0.140	0.070
*FGF23*	Duodenum	5.7	6.7	6.6	6.2	0.32	0.528	0.376	0.069
	Jejunum	ND	ND	ND	ND	-	-	-	-
	Ileum	5.8	6.6	6.2	6.7	0.95	0.819	0.522	0.882
	Cecum	5.9	6.2	6.3	6.2	0.19	0.397	0.752	0.443
	Colon	6.3	6.0	6.5	6.7	0.17	0.026	0.975	0.131
*SLC34A1*	Duodenum	5.9	7.0	6.6	6.9	0.33	0.366	0.091	0.259
	Jejunum	ND	ND	ND	ND	-	-	-	-
	Ileum	ND	ND	ND	ND	-	-	-	-
	Cecum	ND	ND	ND	ND	-	-	-	-
	Colon	ND	ND	ND	ND	-	-	-	-
*SLC34A2*	Duodenum	5.1	7.0	6.6	6.9	0.33	0.366	0.091	0.259
	Jejunum	ND	ND	ND	ND	-	-	-	-
	Ileum	5.7	6.8	5.8	5.9	0.79	0.675	0.550	0.726
	Cecum	6.2	6.0	6.5	6.6	0.17	0.031	0.742	0.540
	Colon	6.8	6.6	6.7	6.9	0.31	0.786	0.961	0.618
*SLC34A3*	Duodenum	6.7	7.0	7.3	7.4	0.31	0.124	0.555	0.940
	Jejunum	7.8	8.1	7.8	8.2	0.30	0.903	0.225	0.929
	Ileum	7.2	6.5	7.0	7.0	0.72	0.875	0.609	0.607
	Cecum	7.0	6.7	6.9	7.2	0.12	0.158	1.000	0.116
	Colon	ND	ND	ND	ND	-	-	-	-

SEM, standard error of the means; ND, not detectable.

1) *VDR*, vitamin D receptor; *CYP24A1*, cytochrom P450, family 24, subfamily A, polypeptide 1 (24-hydroxylase); *TRPV5*, transient receptor potential vanilloid-5; *TRPV6*, transient receptor potential vanilloid-6; *CALB1*, calbindin; *PMCA1b*, plasma membrane Ca^2+^ adenosintriphosphatase 1b; *FGF23*, fibroblast growth factor; *SLC34A1*, Na^+^-Pi cotransporter 2a; *SLC34A2*, Na^+^-Pi cotransporter 2b, *SLC34A3*, Na^+^-Pi cotransporter 2c.

2) A significant gut site effect (p<0.05) was analyzed for the gene expression of *VDR*, *TRPV5*, *TRPV6*, *CALB1*, *PMCA1b*, *SLC34A2*, and *SLC34A3*.

a,b Different superscript letters indicate significant differences between groups at p<0.05.

**Table 7:** Effect of dietary phytase on gene expression related to barrier function along the intestinal tract of fattening pigs (log_10_ copies cDNA/g sample) on experimental days 50 and 51 (n = 12 per diet; 6 gilts and 6 barrows)

Gene^1)^	Gut site^2)^	Control diet		Phytase diet		SEM	p-values		
		
		Gilts	Barrows	Gilts	Barrows		Phytase	Sex	Phytase×sex
*CLDN4*	Duodenum	9.4	9.6	10.7	10.8	0.40	0.006	0.731	0.884
	Jejunum	10.3	9.9	10.2	11.0	0.32	0.139	0.585	0.086
	Ileum	8.5	8.2	8.3	9.2	0.55	0.461	0.595	0.290
	Cecum	9.9	9.7	10.3	10.3	0.29	0.099	0.603	0.710
	Colon	10.5	9.7	10.6	10.5	0.20	0.030	0.043	0.099
*OCLN*	Duodenum	8.7	9.1	10.1	10.0	0.44	0.016	0.642	0.572
	Jejunum	9.7	9.5	9.8	10.5	0.34	0.131	0.493	0.260
	Ileum	7.9	7.7	7.8	8.6	0.56	0.515	0.583	0.359
	Cecum	9.4	9.6	9.9	9.9	0.30	0.230	0.691	0.711
	Colon	10.3	9.6	10.2	10.2	0.20	0.243	0.080	0.089
*ZO1*	Duodenum	8.6	9.1	10.2	10.1	0.52	0.018	0.695	0.605
	Jejunum	9.2	9.2	9.1	10.3	0.54	0.299	0.341	0.262
	Ileum	7.0	7.0	6.6	8.0	0.78	0.724	0.409	0.374
	Cecum	9.7	9.9	10.2	10.1	0.37	0.323	0.861	0.717
	Colon	10.6	9.6	10.5	10.5	0.26	0.103	0.067	0.072
*CDH1*	Duodenum	9.4	9.8	10.7	10.7	0.41	0.017	0.638	0.599
	Jejunum	10.0	10.0	10.1	10.7	0.34	0.279	0.330	0.325
	Ileum	8.5	8.5	8.3	9.1	0.53	0.693	0.493	0.416
	Cecum	10.5	10.5	10.8	10.7	0.25	0.418	0.861	0.836
	Colon	11.2	10.3	11.1	11.1	0.21	0.110	0.043	0.053

SEM, standard error of the means.

1) *CLDN4*, claudin-4; *OCLN*, occludin; *ZO1*, zonula occludens-1; *CDH1*, cadherin-1.

2) A significant gut site effect (p<0.05) was analyzed for the gene expression of *CLDN4*, *OCLN*, *ZO1*, and *CDH1*.

**Table 8:** Effect of dietary phytase on relative gene expression in the kidney of fattening pigs (log_10_ copies cDNA/g sample) on experimental days 50 and 51 (n = 12 per diet; 6 gilts and 6 barrows)

Gene^1)^	Control diet		Phytase diet		SEM	p-values		
		
	Gilts	Barrows	Gilts	Barrows		Phytase	Sex	Phytase×sex
*CYP24A1*	9.6	9.2	9.6	9.8	0.25	0.271	0.697	0.255
*TRPV5*	9.5	9.1	9.3	9.2	0.09	0.842	0.016	0.123
*TRPV6*	6.8	7.2	7.0	6.8	0.26	0.667	0.852	0.293
*CALB1*	10.8	10.6	10.8	10.6	0.13	0.735	0.186	0.969
*PMCA1b*	9.0	9.0	9.0	9.1	0.09	0.832	0.515	0.758
*FGF23*	5.8	5.7	5.8	5.7	0.07	0.698	0.104	0.611
*SLC34A1*	11.7	11.7	11.7	11.6	0.08	0.747	0.394	0.637
*SLC34A2*	6.4	6.5	6.5	6.4	0.15	0.884	0.816	0.702
*SLC34A3*	9.1	8.9	9.1	8.7	0.12	0.509	0.050	0.517

SEM, standard error of the means.

1) *CYP24A1*, cytochrom P450, family 24, subfamily A, polypeptide 1 (24-hydroxylase); *TRPV5*, transient receptor potential vanilloid-5; *TRPV6*, transient receptor potential vanilloid-6; *CALB1*, calbindin; *PMCA1b*, plasma membrane Ca^2+^ adenosintriphosphatase; *FGF23*, fibroblast growth factor 23; *SLC34A1*, Na^+^-Pi cotransporter 2a; *SLC34A2*, Na+-Pi cotransporter 2b; *SLC34A3*, Na^+^-Pi cotransporter 2c.

**Table 9:** Pearson’s correlation between serum parameters, metacarpal bone parameters, and mineral intake on experimental days 50 and 51 (n = 24 per parameter; |r|>0.35 and p<0.05 considered as significant correlation)

Items	Serum parameters						Metacarpal bone parameters						Mineral intake^1)^		
		
	Phosphorus (mmol/L)	Calcium (mmol/L)	Calcium/phoshporus ratio	Fibroblast growth factor 23 (pg/mL)	Vitamin D (pg/mL)	Alkaline phosphatase (U/L)	Osteocalcin (ng/mL)	Length (mm)	Weight (g)	Cortical wall thickness (mm)	Cross-sectional area (mm^2^)	Cortical index	Average daily tP intake (g/d)	Average daily aP intake (g/d)	Average daily Ca intake (g/d)
Serum parameters															
Phosphorus (mmol/L)	1														
Calcium (mmol/L)	0.16	1													
Calcium/phosphorus ratio	−0.89*	0.28	1												
FGF23^$^ (pg/mL)	−0.65*	−0.38	0.49*	1											
Vitamin D (pg/mL)	−0.04	−0.36	−0.10	0.37	1										
ALP^+^ (U/L)	0.15	0.44*	0.02	−0.22	−0.18	1									
Osteocalcin (ng/mL)	−0.14	0.18	0.21	0.09	−0.27	0.10	1								
Metacarpal bone parameters															
Length (mm)	0.29	−0.25	−0.36	0.18	0.59*	0.06	−0.30	1							
Weight (g)	−0.03	0.08	0.05	−0.06	−0.29	0.09	−0.08	0.09	1						
Cortical wall thickness (mm)	−0.01	0.00	0.01	−0.03	−0.18	0.14	−0.10	0.34	0.89*	1					
Cross-sectional area (mm^2^)	0.10	0.13	−0.06	0.02	0.19	0.10	−0.14	0.00	−0.37	−0.30	1				
Cortical index	0.12	0.09	−0.12	0.09	0.22	0.13	−0.20	0.14	−0.26	−0.16	0.95*	1			
Mineral intake^1)^															
Average daily tP intake (g/d)	0.05	0.16	0.03	−0.11	0.13	0.06	0.00	−0.17	−0.47*	−0.45*	0.88*	0.68*	1		
Average daily aP intake (g/d)	0.01	0.11	0.02	−0.32	−0.31	0.15	0.05	−0.14	0.02	0.10	0.18	0.12	0.27	1	
Average daily Ca intake (g/d)	−0.11	0.12	0.18	−0.09	−0.11	0.29	0.37	−0.03	0.30	0.33	−0.23	−0.19	−0.20	0.44*	1

Significant correlations with |r|>0.35 and p<0.05 are marked with *.

1) tP, total phosphorus; aP, available phosphorus; Ca, calcium.

## SUPPLEMENTARY MATERIAL

**Supplementary Table S1**. Oligonucleotide primers used for quantitative PCR

**Supplementary Table S2**. Effect of dietary phytase and sex on body weight, average daily feed intake, calcium (Ca) intake, total phosphorus (tP) intake, available phosphorus (aP) intake, average daily gain and feed conversion ratio in fattening pigs used in replicate batches1 and 2 between experimental days 1 and 49 (n = 72 per diet, mean±SEM)

**Supplementary Table S3**. Effect of dietary phytase and sex on serum parameters related to the calcium (Ca) and phosphorous (P) homeostasis in fattening pigs sampled on experimental days 50 and 51 in replicate batches 1 and 2 (n = 72 per diet, mean±SEM)

**Supplementary Table S4**. Pearson’s correlation coefficients between gastric, cecal, and colonic short-chain fatty acids (SCFA) and gene expression of tight- and adherens-junction proteins in duodenum, cecum and colon on experimental days 50 and 51 (n = 24; |r|>0.35 and p<0.05 considered as significant correlation)

**Supplementary Table S5**. Canonical correlation analysis relating serum parameters with bone parameters, mineral intake with serum parameters, and mineral intake with bone parameters on experimental days 50 and 51 (n = 24)

**Supplementary Table S6**. Canonical correlation analysis relating gastric, cecal, and colonic short-chain fatty acids with the gene expression of tight- and adherens-junction proteins in duodenum, cecum and colon on experimental days 50 and 51 (n = 24)
