# Supplementary material for: Available phosphorus levels modulate gene expression related to intestinal calcium and phosphorus absorption and bone parameters differently in gilts and barrows
Source: Anim Biosci. 2022 Nov 14;36(5):740–52. doi: 10.5713/ab.22.0251 (PMC10164474; doi:10.5713/ab.22.0251)
Supplement: Supplementary file 1 [file ab-22-0251-Supplementary-Table-1.pdf]

Available phosphorus levels modulate gene expression related to intestinal calcium and phosphorus absorption and bone parameters differently in gilts and barrows

Julia Christiane *Vötterl*[1], Jutamat *Klinsoda*[2], Simone *Koger*[3], Isabel *Hennig-Pauka*[4], Doris *Verhovsek*[5] and Barbara U. *Metzler-Zebeli*[1*]

*Corresponding Author: Barbara U. Metzler-Zebeli
Tel: +43 1 25077 3209 4117; E-mail: Barbara.Metzler@vetmeduni.ac.at

[1]Nutritional Physiology, Institute of Physiology, Pathophysiology and Biophysics, Department of Biomedical Sciences, University of Veterinary Medicine Vienna, Vienna, Austria

[2]Institute of Food Research and Product Development, University of Kasetsart, Bangkok, Thailand

[3]Institute of Animal Nutrition and Functional Plant Compounds, Department for Farm Animals and Veterinary Public Health, University of Veterinary Medicine Vienna, Vienna, Austria

[4]Field Station for Epidemiology, University of Veterinary Medicine Hannover, Foundation, Bakum, Germany

[5]University Clinic of Swine, Department for Farm Animals and Veterinary Public Health, University of Veterinary Medicine, Vienna, Austria

SUPPLEMENTARY MATERIAL

Supplementary Table S1. Oligonucleotide primers used for quantitative PCR

Genes[1)]	Accession number[2)]	Primer sequence (5’ to 3’)	Amplification efficiencies	Amplicon size (bp)	Reference
ACTG	XM_003357928.1	F: GGGCATCCTGACCCTCAAG	96.1	89	[1]
		R: TGTAGAAGGTGTGATGCCAGATCT			
GAPDH	NM_001206359.1	F: GGCGTGAACCATGAGAAGTATG	98.7	60	[1]
		R: GGTGCAGGAGGCATTGCT			
B2M	NM_213978.1	F: CCCCCGAAGGTTCAGGTT	100.3	66	[1]
		R: GCAGTTCAGGTAATTTGGCTTTC			
HPRT	NM_001032376.2	F: AGAAAAGTAAGCAGTCAGTTTCATATCAGT	82.8	131	[1]
		R: ATCTGAACAAGAGAGAAAATACAGTCAATAG			
OAZ1	NM_001122994.1	F: TCGGCTGAATGTAACAGAGGAA	94.7	70	[1]
		R: GAGCCTGGATTGGACGTTTAAA			
VDR	NM_001097414.1	F: TGGTTGGAAGTGTCTGGGAG	99.8	117	[2]
		R: GGGGTCAGGTAAGGAAGTGC			
CYP24A1	NM_214075.2	F: TTGGGTTCGTTCGACTCCG	97.2	103	[2]
		R: TCCACGGTTTGATCTCCAGC			

		R:	ATAACTGTTTAGCCAGCAGCAC			
TRPV5	XM_021078896.1,	F:	TCCCTGTAACTTGCCAGTGC	94.2	103	[2]
	XR_002340352.1	R:	TGCTGATCCCAGTCTTGCTG			
TRPV6	FJ268731.2	F:	GAATGCGGTTGCATTGAGCA	97.6	112	[2]
		R:	TTACACCCTTTCCACAGCCG			
CALB1	NM_001130226.1	F:	ATTTCGACGCTGACGGAAGT	91.7	224	[2]
		R:	TTGCTGGCATCGGAATAGCA			
PMCA1b	X53456.1,	F:	GAAAATGGTTCCTGCTGCC	92.3	275	[2]
	XR_002343820.1,	R:	GCAACCGAGTTGTTTGCCAT			
	XM_021091182.1,					
	NM_214352.3	R:	GCAACCGAGTTGTTTGCCAT			
FGF23	XM_001926525.4	F:	CGCAGGCTTCGTGGTCATAA	99.0	146	[2]
		R:	GGTACACGTCGTAGCCGTTC			
SLC34A1	NM_001044623.1	F:	TCAACTCTCTGCTCAAGGGC	94.0	183	[2]
		R:	CACCTAGGCCAATGAGTGGG			
SLC34A2	NM_001256772.1	F:	CGTGTTCTCGTCGACTCTGA	90.0	280	[2]
		R:	CCAGCGGTACTTGGATGAGAT			

SLC34A3	XM_021081180.1	F:	CTTGATGGGGGCTCCAGAC	93.0	98	Newly designed
		R:	CCACCCTCAGTGATGCAGAG			
CLDN4	NM 001161637.1	F:	CAACTGCGTGGATGATGAGA	90.3	140	[3]
		R:	CCAGGGGATTGTAGAAGTCG			
ZO1	XM_003353439.2	F:	AAGCCCTAAGTTCAATCACAATCT	100.6	130	[3]
		R:	ATCAAACTCAGGAGGCGGC			
		R:	GAGCCTGGATTGGACGTTTAAA			
CDH1	NM_001163060.1	F:	TACCTGAACGAGTGGGGCAA	94.9	118	[3]
		R:	CCCATCACATGAGCGTAGGG			

F, forward primer; R, reverse primer.

[1)] *ACTG*, β-actin; *GAPDH*, glyceraldehyde-3-phosphate-dehydrogenase; *B2M*, β2-microglobulin; *HPRT*, hypoxanthin-guanine phosphoribosyl transferase; *OAZ1*, ornithine decarboxylase antizyme; *VDR*, vitamin D receptor; *CYP24A1*, cytochrome P450, family 24, subfamily A, polypeptide 1; *TRPV5*, transient receptor potential vanilloid 5; *TRPV6*, transient receptor potential vanilloid 6; *CALB1*, calbindin; *PMCA1b*, plasma membrane Ca^{2+} adenosintriphosphatase 1b; *FGF23*, fibroblast growth factor 23; *SLC34A1*, Na+-Pi cotransporter 1; *SLC34A2*, Na+-Pi cotransporter 2; *SLC34A3*, Na+-Pi cotransporter 3; *CLDN4*, claudin-4; *OCLN*, occludin; *ZO1*, zonula occludens-1; *CDH1*, cadherin-1.

[2)] National Center for Biotechnology Information (NCBI) (http://www.ncbi.nlm.nih.gov/sites/entrez?db=gene).

1. Metzler-Zebeli BU, Ertl R, Klein D et al. Explorative study of metabolic adaptations to various dietary calcium intakes and cereal sources on serum metabolome and hepatic gene expression in juvenile pigs. Metabolomics 2015;11:545–558. x

2. Vötterl JC, Klinsoda J, Zebeli Q et al. Dietary phytase and lactic acid-treated cereal grains differently affected calcium and phosphorus homeostasis from intestinal uptake to systemic metabolism in a pig model. Nutrients 2020;12:1542. https://doi.org/10.3390/nu12051542

3. Klinsoda J, Vötterl J, Zebeli Q et al. Alterations of the viable ileal microbiota of gut-mucosa-lymph node axis in pigs fed phytase and lactic acid-treated cereals. Appl Environ Microbiol 2020;86:e02128-19. https://doi.org/10.1128/AEM.02128-19
