# Supplementary material for: Available phosphorus levels modulate gene expression related to intestinal calcium and phosphorus absorption and bone parameters differently in gilts and barrows
Source: Anim Biosci. 2022 Nov 14;36(5):740–52. doi: 10.5713/ab.22.0251 (PMC10164474; doi:10.5713/ab.22.0251)
Supplement: Supplementary file 2 [file ab-22-0251-Supplementary-Table-2.pdf]

Supplementary Table S2. Effect of dietary phytase and sex on body weight, average daily feed intake, calcium intake, total phosphorus intake, available phosphorus intake, average daily gain and feed conversion ratio in fattening pigs used in replicate batches1 and 2 between experimental days 1 and 49 (n = 72 per diet, mean ± SEM)

	Control diet		Phytase diet			p-Values		
	Gilts	Barrows	Gilts	Barrows	SEM	Phytase	Sex	Phytase × Sex
n per dietary group	37	35	38	34				
Body weight day 1 (kg)	35.5	39.0	38.3	38.9	0.99	0.155	0.041	0.151
Body weight day 49 (kg)	83.2	85.2	80.7	83.8	1.18	0.100	0.043	0.705
Average daily feed intake (kg/d)	2.02	2.31	1.86	2.12	0.038	<0.001	<0.001	0.792
Ca[1)] intake (g/d)	16.4	19.6	15.9	18.0	0.29	<0.001	<0.001	0.079
Total P[2)] intake (g/d)	12.5	14.9	11.8	13.4	0.22	<0.001	<0.001	0.067
Available P intake (g/d)	6.47	7.71	7.81	8.84	0.125	<0.001	<0.001	0.404
Average daily gain (kg/d)	0.94	0.98	0.89	0.95	0.025	0.098	0.042	0.739
Feed conversion ratio	2.17	2.36	2.13	2.26	0.033	0.032	<0.001	0.425

SEM, standard error of the means.

[1)] Ca, calcium.

[2)] P, phosphorus.
