# Supplementary material for: Available phosphorus levels modulate gene expression related to intestinal calcium and phosphorus absorption and bone parameters differently in gilts and barrows
Source: Anim Biosci. 2022 Nov 14;36(5):740–52. doi: 10.5713/ab.22.0251 (PMC10164474; doi:10.5713/ab.22.0251)
Supplement: Supplementary file 3 [file ab-22-0251-Supplementary-Table-3.pdf]

Supplementary Table S3. Effect of dietary phytase and sex on serum parameters related to the calcium (Ca) and phosphorous (P) homeostasis in fattening pigs sampled on experimental days 50 and 51 in replicate batches 1 and 2 (n = 72 per diet, mean ± SEM)

	Control diet		Phytase diet			p-Values		
Serum parameters[1)]	Gilts	Barrows	Gilts	Barrows	SEM	Phytase	Sex	Phytase × Sex
Phosphorus (mmol/ L)	2.89	2.91	2.80	2.76	0.042	0.004	0.840	0.421
Calcium (mmol/ L)	2.56	2.59	2.57	2.54	0.018	0.481	0.924	0.091
Ca/P ratio	0.89	0.89	0.92	0.93	0.015	0.031	0.751	0.897
FGF23 (pg/ mL)	857	759	888	978	61.8	0.043	0.949	0.126
VitD (pg/ mL)	16.0	17.1	17.6	19.4	0.77	0.014	0.059	0.647
ALP (U/ L)	138	137	153	148	6.4	0.040	0.599	0.723
Osteocalcin (ng/ mL)	25.6	26.5	27.2	28.3	1.22	0.161	0.415	0.956
Urea (mg/ dL)	17.6	22.8	19.4	24.3	1.19	0.168	<0.001	0.899
Cholesterol (mg/ dL)	100.6	103.7	104.6	103.4	2.375	0.436	0.700	0.367
Triglyceride (mg/ dL)	28.9	32.3	35.0	33.6	1.548	0.017	0.526	0.120
NEFA (mmol/ L)	0.26	0.26	0.40	0.40	0.039	0.001	0.979	0.918

SEM, standard error of the means.

[1)] Ca/P ratio, calcium to phosphorus ratio; FGF23, fibroblast growth factor 23; VitD, 25-hydroxyvitamin D_3 and D_2; ALP, alkaline phosphatase; NEFA, non-esterified fatty acids.
