# Supplementary material for: Available phosphorus levels modulate gene expression related to intestinal calcium and phosphorus absorption and bone parameters differently in gilts and barrows
Source: Anim Biosci. 2022 Nov 14;36(5):740–52. doi: 10.5713/ab.22.0251 (PMC10164474; doi:10.5713/ab.22.0251)
Supplement: Supplementary file 4 [file ab-22-0251-Supplementary-Table-4.pdf]

Supplementary Table S4. Pearson's correlation coefficients between gastric, cecal, and colonic short-chain fatty acids and gene expression of tight- and adherens-junction proteins in duodenum, cecum and colon on experimental days 50 and 51 (n = 24; ǀrǀ > 0.35 and p < 0.05 considered as significant correlation)

			Short-chain fatty acids (µmol/g)								
			Acetate	Propionate	Isobutyrate	Butyrate	Valerate	Isovalerate	Caproate	Heptanoate	Total SCFA
Gene expression (log10 copies cDNA/g sample)[1)]	Stomach/ Duodenum	*CDH1*	0.13	-0.29	-0.04	-0.17	-0.40	-	0.15	-	0.11
		CLDN4	0.13	-0.38	-0.01	-0.24	-0.47*	-	0.20	-	0.11
		OCLN	0.11	-0.33	-0.03	-0.20	-0.45*	-	0.19	-	0.09
		ZO1	0.08	-0.36	-0.03	-0.23	-0.44*	-	0.13	-	0.06
	Cecum	*CDH1*	0.29	0.19	-0.05	0.28	-0.11	-0.25	-0.14	-0.40	0.28
		CLDN4	0.31	-0.08	0.18	-0.02	0.11	-0.50*	-0.28	-0.46*	0.12
		OCLN	0.30	0.17	0.01	0.19	-0.05	-0.22	-0.16	-0.35	0.27
		ZO1	0.32	0.21	-0.02	0.25	-0.09	-0.21	-0.12	-0.35	0.31
	Colon	*CDH1*	0.16	0.05	0.10	-0.02	0.09	-0.10	0.06	-0.04	0.11
		CLDN4	0.17	-0.03	0.24	-0.10	0.21	-0.09	-0.08	0.06	0.09
		OCLN	-0.09	-0.16	0.19	-0.24	0.18	-0.13	0.03	-0.21	-0.12
		ZO1	0.00	-0.12	0.16	-0.18	0.14	-0.21	-0.04	-0.15	-0.05

Significant correlations with ǀrǀ > 0.35 and p < 0.05 are marked with *.

[1)] *CDH1*, cadherin-1; *CLDN4*, claudin-4; *OCLN*, occludin; *ZO1*, zonula occludens-1.
