# Supplementary material for: Available phosphorus levels modulate gene expression related to intestinal calcium and phosphorus absorption and bone parameters differently in gilts and barrows
Source: Anim Biosci. 2022 Nov 14;36(5):740–52. doi: 10.5713/ab.22.0251 (PMC10164474; doi:10.5713/ab.22.0251)
Supplement: Supplementary file 5 [file ab-22-0251-Supplementary-Table-5.pdf]

Supplementary Table S5. Canonical correlation analysis relating serum parameters with bone parameters, mineral intake with serum parameters, and mineral intake with bone parameters on experimental days 50 and 51 (n = 24).

Correlated data sets	Canonical function	Canonical correlation	Approximate F value	p-Values
Serum parameters[1)] with bone parameters[2)]	1	0.954	1.24	0.252
	2	0.845	0.76	0.802
	3	0.715	0.54	0.945
	4	0.588	0.39	0.971
	5	0.328	0.19	0.982
Mineral intake[3)] with serum parameters	1	0.764	0.71	0.827
	2	0.635	0.56	0.900
	3	0.515	0.48	0.860
Mineral intake with bone parameters	1	0.812	2.04	0.033
	2	0.498	0.86	0.562
	3	0.280	0.51	0.681

[1)] Serum parameters include the data for phosphate (mmol/L), calcium (mmol/L), calcium to phosphate ratio, fibroblast growth factor 23 (pg/mL), vitamin D (ng/mL), alkaline phosphatase (U/L), osteocalcin (ng/mL), urea (mg/dL), cholesterol (mg/dL), triglycerides /mg/dL) and non-esterified fatty acids (mmol/L).

[2)] Bone parameters include the data for length, weight, cortical wall thickness, cross-sectional area and cortical index of metacarpal bone.

[3)] Mineral intake include the data for total phosphorus intake, available phosphorus intake and calcium intake.
