# Supplementary material for: Available phosphorus levels modulate gene expression related to intestinal calcium and phosphorus absorption and bone parameters differently in gilts and barrows
Source: Anim Biosci. 2022 Nov 14;36(5):740–52. doi: 10.5713/ab.22.0251 (PMC10164474; doi:10.5713/ab.22.0251)
Supplement: Supplementary file 6 [file ab-22-0251-Supplementary-Table-6.pdf]

Supplementary Table S6. Canonical correlation analysis relating gastric, cecal, and colonic short-chain fatty acids with the expression of tight- and adherens-junction proteins in duodenum, cecum and colon on experimental days 50 and 51 (n = 24).

Correlated data sets		Canonical function	Canonical correlation	Approximate F value	p-Values
Short-chain fatty acids[1)] with relative gene expression of tight-and adherens-junction proteins[2)]	Stomach	1	0.802	1.68	0.062
		2	0.746	1.26	0.270
		3	0.424	0.45	0.878
		4	0.129	0.10	0.961
	Cecum	1	0.854	1.54	0.090
		2	0.800	1.11	0.378
		3	0.511	0.45	0.924
		4	0.228	0.17	0.972
	Colon	1	0.862	1.83	0.030
		2	0.722	1.43	0.166
		3	0.642	1.40	0.224
		4	0.579	1.51	0.244

[1)]Short.-chain fatty acids (µmol/g) include the data for acetate, propionate, isobutyrate, butyare, isovalerate, valerate, caproate, heptanoate and total short-chain fatty acids.

[2)]The relative gene expression of tight- and adherens-junction proteins include the data for claudin-4, occludin, zonula occludens-1 and cadherin-1.
